# Systematic review and meta-analysis of contemporary pancreas surgery with arterial resection

**DOI:** 10.1007/s00423-020-01972-2

**Published:** 2020-09-07

**Authors:** Artur Rebelo, Ibrahim Büdeyri, Max Heckler, Jumber Partsakhashvili, Jörg Ukkat, Ulrich Ronellenfitsch, Christoph W. Michalski, Jörg Kleeff

**Affiliations:** 1 Department of Visceral, Vascular and Endocrine Surgery, University Hospital Halle (Saale), Martin-Luther-University Halle-Wittenberg, Ernst-Grube-Str. 40, 06120 Halle (Saale), Germany; 2 Department for Cancer Immunology and Virology, Dana Farber Cancer Institute, Boston, MA USA

**Keywords:** Pancreatic surgery, Arterial resection, Vascular, Pancreatic cancer

## Abstract

**Objective:**

Advances in multimodality treatment paralleled increasing numbers of complex pancreatic procedures with major vascular resections. The aim of this meta-analysis was to evaluate the current outcomes of arterial resection (AR) in pancreatic surgery.

**Methods:**

A systematic literature search was carried out from January 2011 until January 2020. MOOSE guidelines were followed. Predefined outcomes were morbidity, pancreatic fistula, postoperative bleeding and delayed gastric emptying, reoperation rate, mortality, hospital stay, R0 resection rate, and lymph node positivity. Duration of surgery, blood loss, and survival were also analyzed.

**Results:**

Eight hundred and forty-one AR patients were identified in a cohort of 7111 patients. Morbidity and mortality rates in these patients were 66.8% and 5.3%, respectively. Seven studies (579 AR patients) were included in the meta-analysis. Overall morbidity (48% vs 39%, *p* = 0.1) and mortality (3.2% vs 1.5%, *p* = 0.27) were not significantly different in the groups with or without AR. R0 was less frequent in the AR group, both in patients without (69% vs 89%, *p* < 0.001) and with neoadjuvant treatment (50% vs 86%, *p* < 0.001). Weighted median survival was shorter in the AR group (18.6 vs 32 months, range 14.8–43.1 months, *p* = 0.037).

**Conclusions:**

Arterial resections increase the complexity of pancreatic surgery, as demonstrated by relevant morbidity and mortality rates. Careful patient selection and multidisciplinary planning remain important.

**Electronic supplementary material:**

The online version of this article (10.1007/s00423-020-01972-2) contains supplementary material, which is available to authorized users.

## Introduction

Surgery for pancreatic cancer has become increasingly safe in the last decades. Complex venous resections are no longer a criterion of unresectability [1, 2], but are a standard addition to the surgical armamentarium in most centers. However, arterial resections in pancreatic cancer surgery have been associated with high morbidity and mortality rates. Mollberg and colleagues [3] reported in their systematic review and meta-analysis from studies published between 1973 and 2010 that median morbidity and mortality rates across these studies were 53.6% and 11.8%, respectively. Consequently, most centers have adopted a restrictive approach for these procedures.

With the advent of effective chemotherapy regimens (namely FOLFIRINOX and nab-paclitaxel/gemcitabine) in the last decade, an increasing number of patients with locally advanced disease at diagnosis now present with a response to neoadjuvant treatment [4, 5]. These patients—most of them considered inoperable 10 years ago—now frequently proceed to resection, and porto-mesenteric venous resections have become routine procedures in high-volume centers in this setting [6, 7]. Arterial resections—albeit to a smaller extent—are performed in selected patients as well.

Technical improvements, including more effective means of hemorrhage control, improved perioperative care, and multidisciplinary approaches including trained vascular surgeons have facilitated this increase. Nonetheless, arterial resections add to the intricacy and morbidity of pancreatic resections. We thus aimed at systematically reviewing and meta-analyzing current perioperative and oncological outcomes of patients who underwent pancreatic cancer surgery with arterial resection.

## Methods

The literature search and data analysis were conducted in accordance with the “meta-analysis for observational studies” (MOOSE) guidelines [8]. The study was prospectively registered in the PROSPERO database (registration number 2019 CRD42019140206; https://www.crd.york.ac.uk/prospero/display_record.php?RecordID=140206).

### Search strategy

The PubMed/Medline, Cochrane Library, CINAHL, ClinicalTrials.gov (clinical trials registry), and WHO ICTRP (clinical trials registry) databases were searched for this study through their respective online search engines. The search was performed on studies published between January 2011 (as the meta-analysis that set the standard on this topic dates back to 2011) [3] and January 2020 (the number of studies including neoadjuvant therapies considerably increased by that time). The optimal literature search for systematic reviews in surgery was followed [9].The search strategies used in the single databases are displayed in Supplementary Table 1. Furthermore, the reference lists of the available studies were manually searched to find relevant articles. The last search was conducted on 09 January 2020. Abstracts and full-text reviews were evaluated independently in an unblinded standardized manner by two authors (AR and IB) in order to assess eligibility for inclusion or exclusion. Disagreements between reviewers were resolved by consensus; if no agreement could be reached, a third author (JP) decided if the respective study was to be included.

### Inclusion and exclusion criteria

**Fig. 1:**
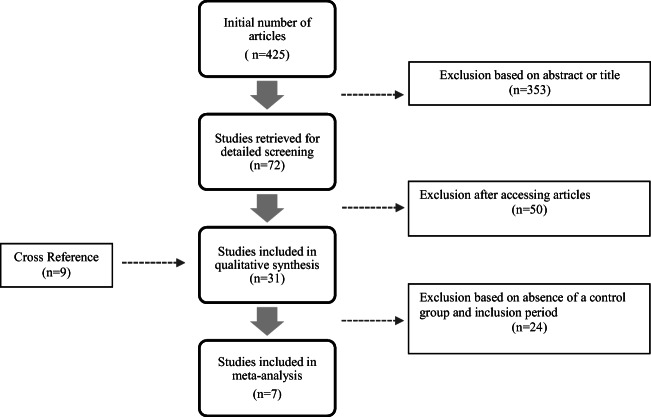
Flowchart with the number of studies identified, screened, assessed, and finally included in the meta-analysis

Studies in English assessing resection of pancreatic cancer in curative intent including resection of major visceral arteries (celiac trunk and/or SMA and/or common hepatic artery), with or without a control group of patients undergoing pancreatic resection without arterial resection, were included. Studies reporting on splenic artery resection were excluded [10]. Studies published before 2011 were also not included, as for example the study by Bockhorn et al. [11]. Studies with an irrelevant abstract or title or with less than five patients were excluded, as were reviews, case reports, comments, and letters. Studies with no differentiation between venous and arterial resection were also excluded. When studies from the same authors including the same patient population were found, the study with a control group was selected. When no control group was described, the studies with fewer patients were excluded. The studies by Klompmaker et al. [12, 13] were excluded as they included data from other single-center studies which were already included in our analysis. Details of the study selection process are summarized in the flowchart of Fig. 1.

After performing a qualitative analysis, only studies including only patients operated from 2000 on with a control group regarding standard resection with curative intention were selected for meta-analysis.

### Data collection

Studies were analyzed and data was extracted separately by two authors (AR and IB) and presented in a tabular fashion (Tables 1, 2, and 3). The following descriptive data was documented for each selected study: first author, year of publication, inclusion period, sample size, arterial resection, neoadjuvant therapy, country, and median follow-up (Table 1). The following patient and operation characteristics were documented: age, gender, type of operation, type of artery resected, duration of surgery, and blood loss (Table 2). The following clinically relevant outcomes for pancreatic surgery were also extracted: morbidity (any type of complication, surgical and medical), pancreatic fistula [45], delayed gastric emptying [46], postoperative bleeding (International Study Group of Pancreatic Surgery definitions) [47], reoperation rate, mortality, length of hospital stay, median survival, actuarial survival (1, 2, 3, and 5 years), R0 resection rate (if possible using the Royal College of Pathologists definition [48]), histologic arterial invasion, and lymph node positivity (Table 3). Risk of bias was accessed using the ROBINS-I tool (risk of bias in nonrandomized studies of interventions) [49] (Table 4). No relevant articles in languages other than English were found. No contact with authors was made.

**Table 1:** Characteristics of included studies

Reference	Year	Inclusion period	Sample size (AR/no AR)	AR neoadjuvant therapy (+/−)	Country	Follow-up (months)
Addeo et al. [14]	2015	2007–2012	24/21	24/0	USA/France	–
Amano et al. [15]	2015	2012–2013	13/0	13/0	Japan	14.5
Bachellier et al. [16]	2018	1990–2017	118/0	89/29	France	15.77
Baumgartner et al. [17]	2012	2007–2010	11/0	11/0	USA	–
Beane et al. [18]	2015	2011–2012	20/172	3/17	USA	–
Cesaretti et al. [19]	2016	1998–2015	5/0	5/0	France	–
Christians et al. [20]	2014	2011–2013	10/0	10/0	USA	21
Del Chiaro et al. [21]	2019	2008–2017	34	17/17	Sweden	–
Glebova et al. [22]	2016	1970–2014	35/5591	6/29	USA	17
Ham et al. [23]	2015	2000–2014	7/31	0/7	Korea	–
Jing et al. [24]	201	2005–2010	24/0	–	China	12.67
Loveday et al. [25]	2018	2009–2016	20/11	18/2	Canada	12.6
Miura et al. [26]	2014	1998–2018	50/0	0/50	Japan	45.3
Miyazaki et al. [27]	2017	1999–2015	21/0	9/12	Japan	11
Nakamura et al. [28]	2016	1998–2015	80/0	2/78	Japan	53.5
Ocuin et al. [29]	2016	2007–2015	30	27/1	USA	33
Okada et al. [30]	2013	2005–2010	16/36	0/16	Japan	25
Perinel et al. [31]	2016	2008–2014	14/97	4/10	France	–
Peters et al. [32]	2016	2004–2016	17/51	15/2	Netherlands	8
Rehders et al. [33]	2012	1998–2010	4/104	–	Germany	–
Sakuraba et al. [34]	2012	1998–2010	7/0	–	Japan	–
Sato et al. [35]	2016	2011–2014	17/0	2/15	Japan	14.4
Sugiura et al. [36]	2017	2002–2014	16/71	0/16	Japan	36
Takahashi et al. [37]	2011	1993–2010	16/27	0/16	Japan	15
Tanaka et al. [38]	2012	1998–2007	42/0	–	Japan	–
Tee et al. [39]	2018	1990–2017	111/0	65/46	USA	19
Ueda et al. [40]	2019	2004–2015	31/0	24/7	Japan	–
Wang et al. [41]	2014	2003–2012	15/0	–	China	–
Yamamoto et al. [42]	2012	1991–2009	13/58	–	Japan	18
Yoshitomi et al. [43]	2019	2010–2016	38/0	31/7	Japan	29.6/15.6
Zhou et al. [44]	2014	2006–2013	12/0	9/3	China	–
Overall	2011–2019	1970–2017	841/6270	364/363	9	–

*AR*, arterial resection; *+*, patients having neoadjuvant chemotherapy; *−*, patients without neoadjuvant chemotherapy

**Table 2:** Patient and operation characteristics

Reference	Group	Age (median)	Sex (M/F)	Type of operation	Artery resected	Duration of surgery (min)	Blood loss (mL)
Addeo et al. [14]	AR	–	–	PD (13), TP (2), DP (9)	SMA (9), CA (12), CHA (1), SP (1), LHA (1)	–	–
	No AR	–	–	–	–	–	–
Amano et al. [15]	AR	64.1	10/3	PD (3), TP (4), DP (6)	CA (10), CHA (7)	567 ± 132 (376–862)	1311 ± 64 (860–2480)
Bachellier et al. [16]	AR	62	61/57	PD (51), TP (18), DP (49)	CA (50), HA (29), SMA (35), others (4)	600 (295–1145)	–
Baumgartner et al. [17]	AR	61	5/6	DP (11)	CA (11)	494	700 (500–4500)
Beane et al. [18]	AR	54	6/14	DP (20)	CA (20)	276 (164–617)	–
	No AR	66	57/115	DP (172)	–	207 (66–581)	–
Cesaretti et al. [19]	AR	56.4	–	DP (5)	CA (5)	550 (450–655)	370 (300–600)
Christians et al. [20]	AR	62	4/6	DP (6), CP (1), PD (2), TP (1)	CA (7), CHA (3)	417 (298–574)	800 (300–2500)
Del Chiaro et al. [21]	AR	65	19/15	PD (9). TP (23), DP (2)	CA (32), SMA (3)	426 ± 14	613 ± 72
Glebova et al. [22]	AR	58	21/14	PD (18), TP (1), PPPD (3), NS (3), DP (9), laparoscopic PD (1)	CA (15), CHA (21)	319 ± 40	1285 ± 276
	No AR	63	2281/3310	PD (1365), TP (90), PPPD (2530), NS (392), DP (1213), laparoscopic PD (1)	–	355 ± 34	828 ± 220
Ham et al. [23]	AR	58	3/4	DP (7)	CA (7)	354 (307–520)	727, *p* = 0.024
	No AR	67.5	16/15	DP (31)	–	286 (157–502)	300, *p* = 0.024
Jing et al. [24]	AR	54.5	18/6	DP	CA (24)	200 ± 68	1779 ± 1934
Loveday et al. [25]	AR	57	17/40	PD (16), DP (2), TP (2)	SMA (10), CA and CHA (10)	681 (448–960)	1600 (500–2500)
	No AR			PD (11)	–	563 (405–660)	575 (350–1300)
Miura et al. [26]	AR	64	26/24	DP (50)	CA (50)	454 (306–1037)	940 (420–15,970)
Miyazaki et al. [27]	AR	66	9/12	PD (17), TP (4)	CHA (21)	2290 (740–19,895)	522 (390–774)
Nakamura et al. [28]	AR	65	40/40	DP (80)	CA (80)	436 (248–1037)	880 (162–15,970)
Ocuin et al. [29]	AR	61.9	17/13	DP (30)	CA (30)	430.8 ± 229.9	1552.5 ± 1565.6
Okada et al. [30]	AR	63	11/5	DP (16)	CA (16)	298 (212–465)	1165 (410–2240)
	No AR	68	23/13	DP (36)	–	203 (128–276)	700 (10–2850)
Perinel et al. [31]	AR	65	9/6	PD (3), TP (9), DP (2)	SMA (6), CHA (4), CA (2), RRHA (2)	380 ± 75	826 ± 415
	Standard	67	36/30	PD (44), TP (10), DP (12)	–	290 ± 75	428 ± 428
	VR	67	20/11	PD (20), TP (10), DP (1)	–	330 ± 45	432 ± 356
Peters et al. [32]	AR	65	9/8	DP (17)	CA (17)	404 (342–480)	900 (400–1000)
	No AR	67	29/22	DP (51)	–	309 (220–370)	525 (300–850)
Rehders et al. [33]	AR	–	–	–	SMA (4)	–	–
	Standard	–	–	–	–	–	–
	VR	–	–	–	–	–	–
Sakuraba et al. [34]	AR	61	0/7	DP (1), PD (6)	CHA (7), CA (1)	–	–
Sato et al. [35]	AR	68	13/4	DP (17)	CA (17)	410 (248–564)	420 (150–1650)
Sugiura et al. [36]	AR	70	10/6	DP (16)	CA (16)	338 (259–507)	902 (461–1893)
	No AR	71	44/32	DP (71)	–	263 (129–557)	460 (76–2716)
Takahashi et al. [37]	AR	65	8/8	DP (16)	CA (16)	237 ± 63	782 ± 82
	No AR	70	10/17	DP (27)	–	203 ± 83	634 ± 85
Tanaka et al. [38]	AR	–	–	DP (42)	CA (42)	478 (311–1037)	1030 (420–15,970)
Tee et al. [39]	AR	62.9	62/49	PD (45), DP (46), TP (20)	CA (49), HA (60), SMA (15), multivessel (15)	472 ± 150	1265 ± 1442
Ueda et al. [40]	AR	62	21/10	DP (31)	CA (31)	334 (175–584)	1270 (305–10,270)
Wang et al. [41]	AR	59.2	8/7	DP (15)	CA (15)	295 (225–420)	1000 (400–2000)
Yamamoto et al. [42]	AR	64	10/3	DP (13)	CA (13)	620 (370–840)	1300 (570–4300)
	No AR	66	39/19	DP (58)	–	360 (220–610)	620 (27–2200)
Yoshitomie et al. [43]	AR (neoadjuvant/no neoadjuvant)	66/62	26/12	DP (38)	CA (38)	350 ± 104/353 ± 87	1274 ± 961/1347 ± 1111
Zhou [44]	AR	52	8/4	DP (12)	CA (12)	330 (280–440)	1200 (800–2400)

*AR*, arterial resection; *PD*, pancreaticoduodenectomy; *DP*, distal pancreatectomy; *TP*, total pancreatectomy; *PPPD*, pylorus-preserving pancreaticoduodenectomy; *NS*, not specified; *SMA*, superior mesenteric artery; *CA*, celiac axis; *HA*, hepatic artery; *CHA*, common hepatic artery; *SP*, splenic artery; *LHA*, left hepatic artery; *RRHA*, replaced right hepatic artery; *M*, male; *F*, female

**Table 3:** Surgical complications, outcomes, and pathology

Reference	Group	Morbidity (%)	Pancreatic fistula (%)	DGE (%)	Postoperative bleeding (%)	Reoperation rate (%)	Mortality (%)	Hospital stay (days)	Median survival (months)	Actuarial survival (years, %)				R0 resection (%)	Histological arterial invasion (%)	Lymph node passivity (%)
										1 year	2 years	3 years	5 years			
Addeo et al. [14]	AR	42	–	–	–	–	–	–	15	61	–	0	–	–	37.5	–
	No AR	24	–	–	–	–	–	–	22	81	–	24.1	–	–	–	–
Amano et al. [15]	AR	61	6	46	–	–	0	49	–	92	–	–	–	92	50 (CA), 0 (CHA)	92
Bachellier et al. [16]	AR	41.5	5.93	–	–	10.1	5.1	22	13.7	59	–	13	11.8	52.4	58	80.5
Baumgartner et al. [17]	AR	45	36	–	8	0	–	9	26	–	–	–	–	–	–	91
Beane et al. [18]	AR	35	10	–	35	10	10	8	–	–	–	–	–	–	–	–
	No AR	36	15	5	–	2	1	6	–	–	–	–	–	–	–	–
Cesaretti et al. [19]	AR	100	80	0	–	0	0	–	24	–	–	60	–	60	–	60
Christians et al. [20]	AR	30	0	0	0	10	0	9	–	–	–	–	–	–	10	–
Del Chiaro et al. [21]	AR	62	27.3	–	–	8.8	2.9	18	–	63.7	–	23.3	23.4	26	44	85
Glebova et al. [22]	AR	89	9	11	6	–	–	13	22	50	–	–	19	57	–	91
	No AR	97	13	13	3	–	–	11	47	58	–	–	11	70	–	83
Ham et al. [23]	AR	100	100	14.3	0	–	0	23	15	100	44.4	–		71	100	57
	No AR	–	68	0	3.2	–	3.2	14.5	25	73.7	55.3	–	–	80.6	–	–
Jing et al. [24]	AR	54	42	25	–	–	0	–	9.25	46	–	4	–	–	–	–
Loveday et al. [25]	AR	65	0	–	–	25	5	11.5	14.8	65	10	–	–	100	–	60
	No AR	73	0	–	–	18	0	10	24.2	82	27	–	–	100	–	55
Miura et al. [26]	AR	54	–	–	–	–	–	39	24.7	80.7	–	32.3	24.3	92	28	72
Miyazaki et al. [27]	AR	81	4.8	9.5	–	–	0	–	11	47.6	–	6.6	6.6	43	57	86
Nakamura et al. [28]	AR	–	58.8	28.8	–	7.5	1.3	38	24.9	81.1	–	56.9	32.7	93	–	62.5
Ocuin et al. [29]	AR	73	43	–	–	7	14	10.7	35	–	–	–	–	80	62	50
Okada et al. [30]	AR	–	18.8	12.5	0	0	0	–	25	81	52	–	–	31	–	–
	No AR	–	27.8	0	2.8	0	0	–	32	81	53	–	–	81	–	–
Perinel et al. [31]	AR	14	0	14	–	0	0	23	–	92	55	17	–	57	–	71
	Standard	29	10	8	–	15	3	27	–	64	51	45	–	74	–	70
	VR	32	3	6	–	6	3	23	–	76	52	33	–	39	–	90
Peters et al. [32]	AR	35	5.9	5.9	5.9	–	0	7	20	75	–	–	–	82	–	41.2
	No AR	24	21.6	7.8	2	–	0	6	19	85.2	–	–	–	92	–	40
Rehders et al. [33]	AR	0	–	–	–	–	–	–	17	–	–	–	–	75	–	50
	Standard	67	–	–	–	–	–	–	27.9	–	–	–	–	86	–	43
	VR	49	–	–	–	–	–	–	23.4	–	–	–	–	83	–	20
Sakuraba et al. [34]	AR	43	–	–	15	–	–	–	–	–	–	–	–	86	–	–
Sato et al. [35]	AR	41	41	12	–	–	0	34	16.9	74	–	45	–	94	–	76
Sugiura et al. [36]	AR	88	44	–	–	–	–	26	17.5	75	–	21.4	–	62	56	88
	No AR	63	41	–	–	–	–	21	43.1	85.2	–	52.9	–	88	–	55
Takahashi et al. [37]	AR	56	31	–	–	12.5	6	38	9.7	42.6	–	25.6	–	56	75	56
	No AR	44	19	–	–	0	0	56	30.9	84.1	–	31.1	–	78	15	44
Tanaka et al. [38]	AR	43	17	12	–	–	–	–	24	–	–	–	25	93	–	–
Tee et al. [39]	AR	54	23	23	17	16	13	–	28.5	–	–	–	–	–	–	–
Ueda et al. [40]	AR	32.3	45.2	–	–	–	6.5	37	23.7	74.2	–	34.4	–	42	45	78
Wang et al. [41]	AR	47	33	–	–	–	–	29.6	19	86.7	–	6.7		100	–	–
Yamamoto et al. [42]	AR	92	62	31	8	–	0	62	20.8	–	25.4	–	–	31	62	–
	No AR	60	45	55	3	–	0	25	21.1	–	45.9	–	–	74	48	–
Yoshitomie et al. [43]	AR (neoadjuvant/no neoadjuvant)	39	29	13	–	–	2.6	32/34	38.6/15.6	79	61	34	–	63	–	21
Zhou et al. [44]	AR	75	25	–	0	–	–	–	10	–	–	–	–	–	–	–

*AR*, arterial resection; *DGE*, delayed gastric emptying; *CA*, celiac axis; *CHA*, common hepatic artery

**Table 4:** The risk of bias was classified into low (+), high (−), and unclear or missing data (?) using ROBINS-I (“Risk of bias in nonrandomized studies—of interventions”) recommended by the Cochrane handbook

Reference	A	B	C	D	E	F	G	H
Beane et al. [18]	–	–	–	?	–	–	–	–
Ham et al. [23]	–	–	–	?	?	–	–	–
Loveday et al. [25]	–	–	+	+	+	+	+	+
Okada et al. [30]	–	–	–	?	+	–	?	–
Perinel et al. [31]	–	–	+	–	–	–	–	–
Peters et al. [32]	–	–	–	?	–	–	–	–
Sugiura et al. [36]	–	–	–	?	+	–	?	–

Risk of bias legend: (A) confounding, (B) selection bias, (C) classification of intervention, (D) intended intervention, (E) missing data, (F) measurement of outcomes, (G) reported result, (H) overall

### Statistical analysis

A meta-analysis was performed with morbidity using the Clavien–Dindo classification [50], perioperative mortality, 1 year survival, R0 resection [48], postoperative pancreatic fistula (defined according to the International Study Group on Pancreatic Fistula) [45], and delayed gastric emptying (DGE) rates (defined by the International Study Group of Pancreatic Surgery) [46] as outcome measures (random-effects model and fixed-effects model) using the Review Manager (RevMan) software, version 5.3 (Cochrane Collaboration, Oxford, UK). The magnitude of the effect estimate was visualized by forest plots. An odds ratio (OR) was calculated for binary data. The 95% confidence interval (CI), heterogeneity, and statistical significance were reported for each outcome. The *χ*^2^ and the Kruskal–Wallis tests were used for the evaluation of statistical significance. *p* < 0.05 was considered to be statistically significant. When the studies did not report mean and standard deviation, these were calculated using the methods described by the guidelines of the Cochrane Collaboration [51] and Hozo et al. [52]. As not all studies report individual patient data or hazard ratios, the survival analysis was performed with weighted rates. Furthermore, a subgroup analysis was performed based on the rate of the patients receiving neoadjuvant chemotherapy. Studies with more than 50% of patients receiving neoadjuvant chemotherapy were included in the neoadjuvant subgroup. No subgroup analysis on planned versus not planned resection and arterial reconstruction versus no reconstruction and arterial resection plus venous resection versus arterial resection alone was performed because no control group was available.

## Results

### Systematic review and combined data analysis

Among the 425 articles, 31 studies from 9 countries were included in the qualitative analysis (Tables 1 and 2). Publication years were from 2011 to 2019. The inclusion periods of patients ranged from 1970 to 2018. Within these 30 studies, a total of 841 patients underwent pancreatic surgery with arterial resection or reconstruction and 6270 patients underwent a procedure without arterial resection.

Among the AR groups from all studies, 50% of patients received neoadjuvant chemotherapy (data from 26/31 studies).

The overall morbidity rate was 66.8% (data from 29/31 studies) in the AR group versus 93% (data from 10/11 studies) in the no AR group (*p* < 0.001). The overall postoperative pancreatic fistula (POPF) rate (all grades combined) was 27% in the AR group (data from 26/31 studies) versus 14% in the no AR group (data from 10/11 studies, *p* < 0.001). Regarding DGE (all grades combined), a rate of 19% in the AR group (data from 15/31 studies) versus 13% in the no AR group (data from 7/11 studies, *p* < 0.001) was observed. Postoperative bleeding (all grades combined) was 12.6% in the AR group (data from 11/31 studies) versus 3% in the no AR group (data from 5/11 studies, *p* < 0.001). A reoperation was required in 11% of patients in the AR group (data from 14/31 studies) versus 4.6% of patients in the no AR group (data from 5/11 studies, *p* < 0.001). No grade differentiation could be performed for POPF, DGE, and postoperative bleeding because stratification was not available in all studies.

Postoperative mortality was 5.3% (data from 19/31 studies) in the AR group versus 1.1% (data from 8/11 studies) in the no AR group (*p* < 0.001).

The R0 resection rate (data from 24/31 studies) was 73% in the AR group versus 80% (data from 9/11 studies) in the no AR group (*p* < 0.001). Again, no clear definitions were present across all studies.

In the AR group, 72% of patients had positive lymph nodes (data from 20/31 studies) versus 82% in the no AR group (data from 7/11 studies, *p* < 0.001). Among 354 patients undergoing arterial resection (data from 13/31 studies), 48.5% had histologically proven arterial invasion.

The weighted median survival was 21.9 months (range 9.5–38.6 months, data from 25/31 studies) in the AR group versus 45.7 months (range 19–47 months, data from 10/11 studies) in the no AR group (*p* = 0.008). The weighted actuarial survival at 1, 2, 3, and 5 years was 80, 44, 29, and 21% (data from 19, 6, 14, and 7/31 studies) versus 59, 50, 42, and 11% in the AR versus no AR group (data from 9, 5, 4, and 1/11 studies, respectively.

### Meta-analysis

As the goal of the meta-analysis was to review contemporary pancreas surgery with arterial resection from the last 20 years, we excluded the studies from Takahashi et al. [37], Glebova et al. [22], and Yamamoto et al. [42] because of the inclusion period starting in 1993, 1970, and 1991, respectively. Moreover, the study from Addeo et al. [14] was not included, as it reports and compares the outcomes of surgical resection of borderline resectable (BL) and locally advanced (LA) “unresectable” pancreatic cancer after neoadjuvant chemotherapy and not directly an arterial resection group with a standard resection group. The study from Rehders et al. [33] was also excluded because it only reported less than five patients who underwent arterial resection. The control group from the Del Chiaro study [21] was excluded because it included patients undergoing palliative surgery.

From the 31 studies, seven cohort studies with a total of 579 patients were included in the meta-analysis. In the risk of bias assessment, only the Loveday et al. [25] study was classified as low risk (Table 3).

As depicted in Table 5, there were 110 patients in the AR group and 469 patients in the control group of the included studies. Thirty-eight percent of the patients who underwent arterial resection received neoadjuvant chemotherapy. Seventy-three percent of the patients in the AR group underwent distal pancreatectomy, 17% pancreaticoduodenectomy, and 10% total pancreatectomy. Ninety-one percent underwent a CA or hepatic artery branches (HAB) resection and 9% an SMA resection. Sixty percent of the arterial resections were planned and only 34% of all patients in the AR group underwent arterial reconstruction.

**Table 5:** Data on selected studies for meta-analysis

Reference	Inclusion period	Sample (AR/no AR)	AR neoadjuvant therapy (+/−)	Operation (AR/no AR)	Artery resected	Reconstruction (Y/N)	Planned resection (Y/U)	Median survival (months) (AR/no AR)	Study type	Risk of bias
Beane et al. [18]	2011–2012	20/172	5/15 −	DP (20)/DP (172)	CA (20)	N	U	–	Prospective	High
Ham et al. [23]	2000–2014	7/31	0/7 −	DP (7)/DP (31)	CA (7)	N	U	15/25	Retrospective	High
Loveday et al. [25]	2009–2016	20/11	18/2 +	DP (2), PD (16), TP (2)/PD (11)	SMA (10), CA and CHA (10)	Y	Y	14.8/24.2	Retrospective	Low
Okada et al. [30]	2005–2010	16/36	0/16 −	DP (16)/DP (36)	CA (16)	N	Y	25/32	Prospective	High
Perinel et al. [31]	2008–2014	14/97	4/10 −	DP (2), PD (3), TP (9)/PD (64), TP (20), DP (13)	CA (2), SMA (6), CHA (4), RRHA (2)	Y	Y	–	Prospective	High
Peters et al. [32]	2004–2016	17/51	15/2 +	DP (17)/DP (51)	CA (17)	U	U	19/20	Prospective	High
Sugiura et al. [36]	2002–2014	16/71	0/16 −	DP (16)/DP (71)	CA (16)	N	Y	17.5/43.1	Retrospective	High
Overall	2000–2016	110/469	42/68	DP (80), PD (19), TP (11)/DP (374), PD (75), TP (20)	CA or HAB (100), SMA (10)	Y (34)	Y (66)	18.6/32 (WMS)	Retrospective (3), prospective (4)	High (6), low (1)

*AR*, arterial resection; *PD*, pancreaticoduodenectomy; *DP*, distal pancreatectomy; *TP*, total pancreatectomy; *SMA*, superior mesenteric artery; *CA*, celiac axis; *HA*, hepatic artery; *CHA*, common hepatic artery; *RRHA*, replaced right hepatic artery; *HAB*, hepatic artery branches; *Y*, yes; *N*, no; *U*, unknown; *WMS*, weighted median survival

**Fig. 2:**
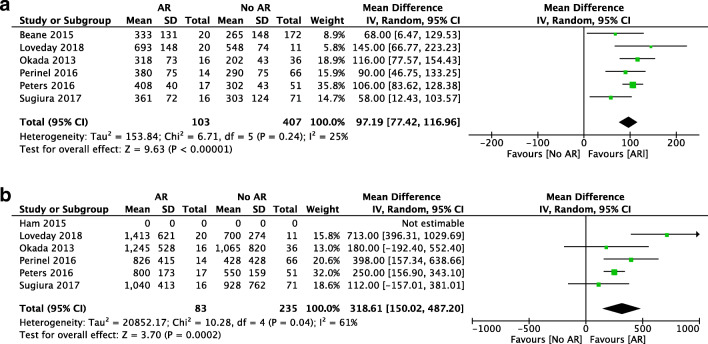
Forest plot for comparing pancreatic surgery with and without arterial resection with regard to operative time (**a**) and blood loss (**b**)

Regarding the duration of the operation, almost all studies demonstrated that pancreatic surgery with AR was longer than standard surgery. In the random-effects model, the operation time was shorter in the standard group with a mean difference of 98 min (95% CI [77.42, 116.96], *p* < 0.001) (Fig. 2a). In all included studies, blood loss was higher in the AR group with a mean difference of 319 mL (95% CI [150.02, 487.2], *p* < 0.001) (Fig. 2b). The study from Ham et al. was excluded as no SD of the mean was provided.

**Fig. 3:**
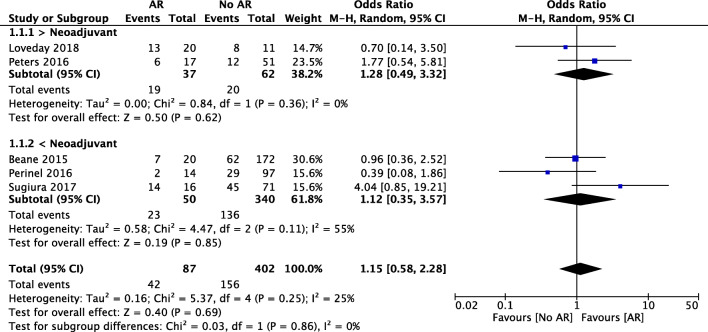
Forest plot for comparing pancreatic surgery with and without arterial resection regarding overall morbidity with subgroup analysis for neoadjuvant therapy

Five studies provided information about overall morbidity. A total of 42 patients who underwent pancreatic surgery with AR were included in this analysis. There was no statistically significant difference between the AR and no AR groups (OR 1.15, 95% CI [0.58, 2.28], *p* = 0.69) (Fig. 3). The overall morbidity rate was 48% in the AR group and 39% in the standard resection group (*p* = 0.1). In the subgroup analysis for neoadjuvant therapy, the results were not significantly different between the neoadjuvant group (OR 1.28, 95% CI [0.49, 3.32]) and the upfront surgery group (OR 1.12, 95% CI [0.35, 3.57], *p* = 0.86).

**Fig. 4:**
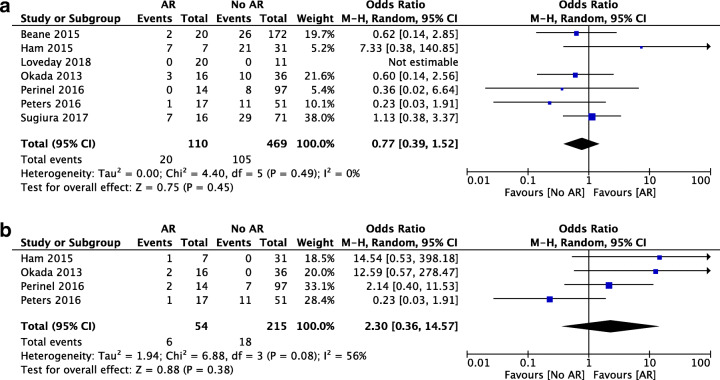
Forest plot for comparing pancreatic surgery with and without arterial resection with regard to postoperative pancreatic fistula (**a**) and delayed gastric emptying (**b**)

Regarding postoperative pancreatic fistula, there was no statistically significant difference in the analysis of 110 patients undergoing pancreatic surgery with AR and 469 undergoing standard surgery (OR 0.77, 95% CI [0.39, 1.52], *p* = 0.45) (Fig. 4a). DGE was assessed in four studies. There was no significant difference in patients receiving AR versus the standard procedure (OR 2.30, 95% CI [0.36, 14.57], *p* = 0.08) (Fig. 4b). Meta-analysis for postoperative bleeding was not performed because this outcome was only reported in two of the selected studies.

**Fig. 5:**
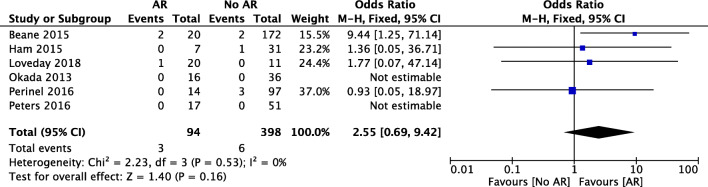
Forest plot for comparing pancreatic surgery with and without arterial resection with regard to mortality

Six of the included studies reported data on perioperative mortality. Mortality was nonsignificantly higher in the AR group (OR 2.55, 95% CI [0.69, 9.42], *p* = 0.16) (Fig. 5). The weighted mortality rate was 3.2% in the AR group and 1.5% in the standard resection group (*p* = 0.27).

**Fig. 6:**
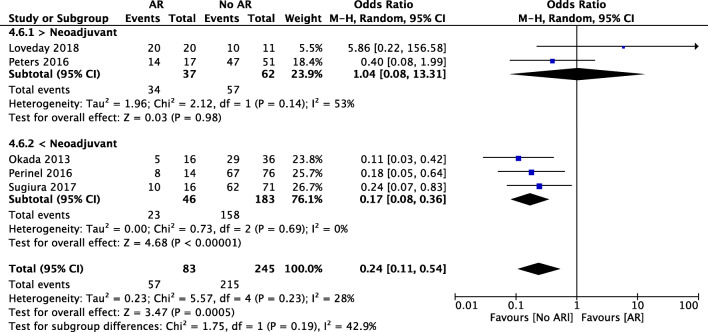
Forest plot for comparing pancreatic surgery with and without arterial resection regarding R0 resection with subgroup analysis of neoadjuvant therapy

Five studies reported on R0/R1 rates although with heterogeneous definitions. Patients undergoing arterial resection had lower R0 resection rates compared to the no AR group (69% vs 89%, OR 0.24, 95% CI [0.11, 0.54], *p* < 0.001). In the subgroup analysis concerning neoadjuvant therapy, patients undergoing upfront surgery with AR showed lower R0 rates when compared with those undergoing standard surgery (50% vs 86%, OR 0.17, 95% CI [0.08, 0.36], *p* < 0.001). Patients undergoing arterial resection after neoadjuvant chemotherapy had no statistically significant difference of R0 rates than the ones undergoing standard resection (92% vs 92%, OR 1.04, 95% CI [0.08, 13.31], *p* = 0.98) (Fig. 6).

**Fig. 7:**
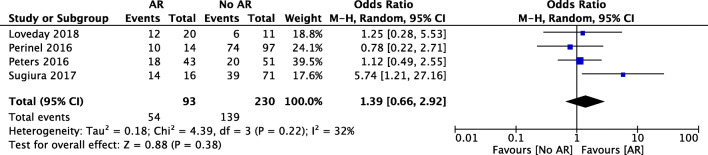
Forest plot for comparing pancreatic surgery with and without arterial resection with regard to lymph node positivity

Four studies were included in the meta-analysis regarding lymph node involvement. Lymph node positivity was observed in 58% of the patients with AR and 60% in the standard group (*p* = 0.69). No significant difference was found in the meta-analysis of the four studies (OR 1.39, 95% CI [0.66, 2.92], *p* = 0.38) (Fig. 7).

In almost all included studies, median survival time was reported. The weighted median survival was 18.6 months (range 14.8–25 months) for patients who underwent pancreatic surgery with AR compared to 32 months (range 19–43.1 months) for patients undergoing a standard procedure without AR (*p* = 0.037). Also, in the neoadjuvant subgroup, the weighted median survival was shorter in patients undergoing AR compared to those undergoing standard resection (16.7 vs 21.3, range 14.8–25 months).

**Fig. 8:**
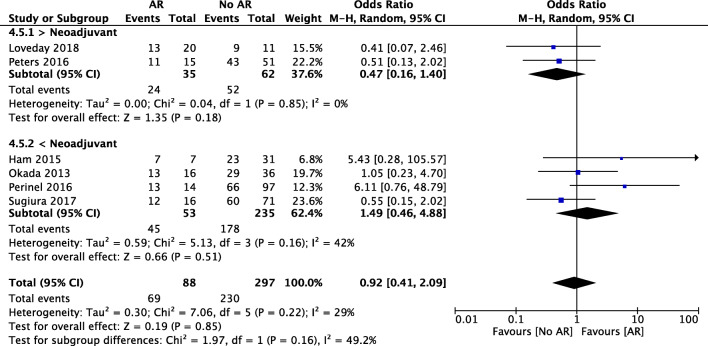
Forest plot for comparing pancreatic surgery with and without arterial resection regarding 1-year survival with subgroup analysis for neoadjuvant therapy

Concerning 1-year survival, the meta-analysis showed no statistically significant difference between the two groups (78% vs 77%, OR 0.92, 95% CI [0.41, 2.09], *p* = 0.85) (Fig. 8). In the subgroup analysis for neoadjuvant therapy, there was no statistical significance neither in the neoadjuvant group (OR 0.47, 95% CI [0.16, 1.40], *p* = 0.18) nor in the upfront surgery group (OR 1.49, 95% CI [0.46, 4.88], *p* = 0.16) between the AR and the no AR groups.

## Discussion

**Fig. 9:**
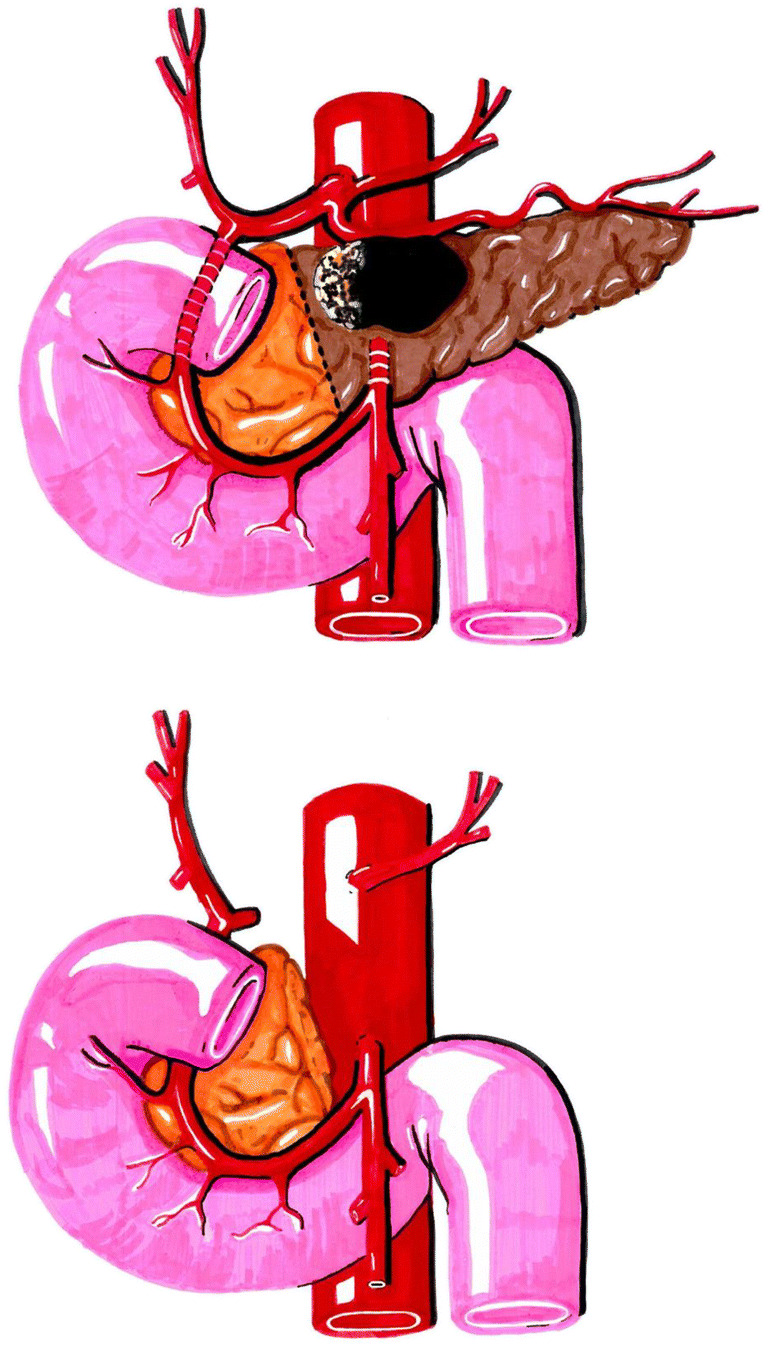
Pancreatic body/tail with CA resection; preservation of the left gastric artery

Arterial resection in pancreatic cancer is strongly related to a borderline or locally advanced, unresectable tumor status. The NCCN guidelines [1] define borderline pancreatic cancer for two locations: pancreatic head/uncinate process with CHA or SMA involvement and pancreatic body/tail with CA involvement (Fig. 9). In the studies included in this meta-analysis, most of the data on arterial resection refers to CA, and most resections were distal pancreatectomies, which carry a lower risk for morbidity and mortality than pancreatic head resections. In the included studies, there was no clear differentiation if arterial resection was performed for borderline resectable, locally advanced, or metastatic tumors, this being one limitation of our analysis.

Another important limitation of our analysis is that six of the seven included studies were classified as having a high risk of bias.

Irrespectively, in our present analysis, there are two major findings. The first concerns comparable postoperative mortality and morbidity rates and the second shorter long-term survival in the arterial resection group.

### Morbidity

In the qualitative analysis, the overall morbidity rate was significantly lower in the AR group. This may be explained by a high morbidity rate of 97% in the standard resection group of the study by Glebova et al. [22]. Here, there were 5591 patients in the control group, which reflects an overrepresentation. In fact, morbidity rates of 41.8–77.5% have been reported in two recent RCTs for major pancreatic resections [53, 54], which is similar to the observed 66.8% morbidity observed in the AR group.

In the meta-analysis, morbidity was not significantly increased in the arterial resection group. This may be attributed to different reasons. One is that the majority of studies in the meta-analysis reported results of the so-called modified Appleby procedure, e.g., distal pancreatectomy with common hepatic artery/celiac trunk resections. Risk of complications is lower in such resections because there is no need for reconstruction—as compared with pancreaticoduodenectomy, for example. In our own experience, morbidity may be significantly increased when performing arterial resections with reconstructions. Different approaches are internationally chosen to these procedures, such as avoiding a pancreatic anastomosis and rather performing total pancreatectomy. Because of this clinical perception, we have designed a multicentric exploratory study for a staged resection in cases with arterial infiltration or arterial stenosis (PREVADER study; protocol currently under review; also, see https://clinicaltrials.gov/ct2/show/NCT04136769). Here, patients with borderline resectable or locally advanced pancreatic ductal adenocarcinoma (PDAC) undergo a first operation—called a visceral debranching procedure—aiming at bypassing the arterial invasion/stenosis, followed by neoadjuvant chemotherapy. If there is no tumor progression (and patency of the arterial bypass) on restaging, patients undergo re-exploratory laparotomy and—if possible—tumor resection. In the second stage, arterial resection can be performed en bloc with the tumor resection without the need of arterial reconstruction.

### Mortality

Unlike the past meta-analysis on this topic [3], we observed no statistically significant difference between the arterial resection and standard surgery groups, although the weighted mortality rate was 3.2% in the arterial resection group and 1.5% in the standard resection group. Possibly, the statistical power of our analysis was not sufficient for such a small absolute difference. Nevertheless, our analysis reports relevantly lower absolute mortality rates compared with those reported by Mollberg et al. of 11.8% [3]. Mortality was differently defined across the studies included in this meta-analysis (in hospital, 30-day and 90-day mortality). Also, these low mortality rates suggest that only highly selected patients were included, which is supported by mortality rates in three recent RCTs of 3–10% [53–55]. The results are comparable to a recent work from Klompmaker et al. [12]. In an analysis of 240 patients, in whom distal pancreatectomy with celiac axis resection (DP-CAR) was combined with (neo-)adjuvant chemotherapy, the 90-day mortality rate was 3.5% [12]. On the other hand, a retrospective cohort study from the same group included 68 patients from 20 hospitals in 12 countries and reported 30-day and 90-day mortality rates after DP-CAR of 10% and 16%, respectively [13].

There is a high risk of bias because patient selection may explain these significant differences between studies and may not reflect the reality even in high-volume centers with less stringent patient selection. Nevertheless, in the light of the presented data, pancreatic surgery with arterial resection can be performed in selected patients with reasonable mortality rates.

Morbidity could not be stratified in grades according to the Clavien–Dindo classification owing to nonavailability of pertinent data. Concerning morbidity, slightly better results could be observed in the standard resection group, but no significant difference was shown. Of note is the significant heterogeneity among the included studies.

Regarding postoperative pancreatic fistula, which is the major cause of morbidity after pancreatic resection, no significant difference was observed between the two groups. It is assumed that most studies reported clinical relevant fistula even though a differentiation of CR-POPF (grade B/C) from POPF-A/biochemical leak was not always made across the included studies. This is supported by CR-POPF rates in three recent RCTs ranging from 6 to 29% as compared with our data of 27% versus 14% [53–55].

Interestingly, lower fistula incidence was reported among the patients with arterial resection, probably due to more advanced tumors and hence more solidified postobstructive fibrosis [56].

Planning the arterial resection may have a positive effect on postoperative morbidity as compared to unplanned resections [31]. Meticulous assessment of preoperative CT scans for detection of possible arterial encasement is therefore essential.

According to our meta-analysis, arterial resection in selected patients does not have a relevant effect on perioperative morbidity, especially on postoperative pancreatic fistula or delayed gastric emptying.

This data has to be interpreted carefully because of the high risk of bias in the included studies as only studies including AR were searched for.

### Long-term survival

The second important aspect emerging from our meta-analysis is that pancreatic surgery with arterial resection is associated with a lower 1-year survival rate than surgery without arterial resection. In a large systematic review and meta-analysis involving 18 studies, DP-CAR had a better 1-year survival rate compared to palliative treatments (pooled HR for OS 0.38 (95% CI 0.25–0.58, *p* < 0.01) [57]. These results are comparable to the weighted average of 1-year survival in our analysis, which was 78% in the AR group. In a study from the Dutch Pancreatic Cancer Group that involved a national cohort of 36,453 patients with PDAC, the 1-year survival of patients who received palliative chemotherapy improved along the last years from 13.3 to 21.2% (*p* < 0.001) [58]. This data is only partial comparable to ours, because this cohort also included patients with advanced metastatic disease (*n* = 4074). According to this data, arterial resection can be considered in selected patients instead of palliative chemotherapy.

Considering weighted median survival in both groups, patients undergoing arterial resection had a worse prognosis with 18.6 months compared with 32 months in patients undergoing surgery without arterial resection. In a recent systematic review that included 240 patients undergoing DP-CAR, the weighted median survival (14.4 months) was comparable to our analysis [12]. Our data are also comparable to data from a multicenter retrospective cohort study regarding patients undergoing arterial resection (18 months median survival) [13].

This finding may be attributed to unfavorable tumor biology with more advanced tumor stages and more aggressive growth in tumors affecting visceral arteries and requiring arterial resection. Longer operative time, lower R0 resection rates, and fewer patients with neoadjuvant treatment in the arterial resection group are additional factors. Nevertheless, arterial resection patients have better prognosis when compared to those undergoing palliative treatments.

In our analysis, despite heterogeneous definitions across the studies, the R0 resection rate was significantly lower in the patients undergoing pancreatic surgery with arterial resection. This can be explained by the local extent of tumor growth and hence the surgical complexity in the arterial resection group. Moreover, neoadjuvant treatment was associated with higher R0 resection rates. Kluger et al. showed in their study that a tumor-free resection margin could be achieved in 80% of the cases after neoadjuvant treatment in locally advanced pancreatic cancer with arterial encasement [59]. In the present series, the weighted average of R0 resection in arterial resection patients within the neoadjuvant subgroup was 92% compared with 50%, in the patients undergoing arterial resection without neoadjuvant chemotherapy (*p* < 0.001). Although neoadjuvant chemotherapy was not associated with prolonged long-term survival, our analysis suggests that it is crucial for achieving negative resection margins in this setting [12, 14, 59]. It has to be emphasized that in the analysis of neoadjuvant therapy, cohorts were grouped according to the predominantly administered therapy. Thus, for example, in the neoadjuvant therapy group, the majority, but not all patients, received neoadjuvant therapy.

The role of alternative therapies like chemoradiotherapy or staged resection needs further investigation. In a phase 3 randomized trial involving 449 patients that underwent either chemotherapy alone or radiochemotherapy, no significant difference between overall survivals of both groups was observed [60]. In a multicenter phase II trial in four hospitals in the Netherlands that enrolled 50 patients, stereotactic body radiotherapy was reported as feasible, and in 12% of the patients, a potentially curative resection could be performed [61]. In another retrospective cohort study, neoadjuvant radiotherapy was associated with increased pathologic downstaging and R0 resection rates [62].

The main drawback of this meta-analysis is that it is based exclusively on nonrandomized and partially retrospective studies. Among these studies, twenty-one had less than 30 patients with arterial resections and only two single-center studies included more than 100 arterial resections. Furthermore, only 34 patients had an arterial resection with arterial reconstruction. The MOOSE guidelines were followed to ensure transparency and standardized reporting, but the risk of bias is still considerable because of the nature of studies included in the meta-analysis. Furthermore, as most of the studies did not report individual patient data or hazard ratios, the survival analysis was performed with weighted rates or weighted median survival which is rather inaccurate surrogate measures for meta-analyses of survival outcomes [63]. Thus, the data should be carefully interpreted.

## Conclusion

In this meta-analysis, all relevant studies published within the last 10 years providing comparative information on the outcome of patients undergoing pancreatic surgery with arterial resection were included. Arterial resections were not associated with significantly higher mortality and morbidity rates. However, probably owing to the more aggressive tumor biology, patients undergoing arterial resection had a shorter survival than patients who did not require arterial resection. Arterial infiltration should not be a strict contraindication against resection in patients with locally advanced disease anymore. Future research should be focused on developing multidisciplinary concepts for these patients. Careful patient selection and treatment planning are mandatory [15]. To address these questions, we have designed an exploratory, multicentric trial to analyze visceral debranching/staged resection combined with neoadjuvant therapy in borderline resectable and locally advanced PDAC (PREVADER study).

## Electronic supplementary material

ESM 1 (DOCX 29 kb)

ESM 2 (DOCX 23.7 kb)
