# Supplementary material for: Systematic review and meta-analysis of contemporary pancreas surgery with arterial resection
Source: Langenbecks Arch Surg. 2020 Sep 7;405(7):903–19. doi: 10.1007/s00423-020-01972-2 (PMC7541389; doi:10.1007/s00423-020-01972-2)
Supplement: Supplementary file 2 — (DOCX 23.7 kb) [file 423_2020_1972_MOESM2_ESM.docx]

## PubMed

| **Number of hits** | **Date** |
| --- | --- |
| 394 | 09.01.2020 |

### P

| **"Pancreatic Neoplasms"[Mesh] OR**  Pancreatic Neoplasm*[tw] OR  Pancreatic Cancer*[tw] OR  Pancreatic Tumor*[tw] OR  Pancreatic Tumour*[tw] OR  Pancreas Neoplasm*[tw] OR  Pancreas Cancer*[tw] OR  Pancreas Tumor*[tw] OR  Pancreas Tumour*[tw] OR  Cancer of Pancreas[tw] OR  Cancer of the Pancreas[tw] |  |
| --- | --- |

### I

| (Resect*[tw] OR  Extirpation[tw] OR  Excision*[tw] OR  ectomy[tw] OR  removal[tw])  AND  **("Hepatic Artery"[Mesh] OR**  Hepatic Arter*[tw] OR  **"Mesenteric Artery, Superior"[Mesh] OR**  Superior Mesenteric Arter*[tw] OR  **"Celiac Artery"[Mesh] OR**  Celiac Arter*[tw]) |  |
| --- | --- |

## Cochrane Library

| **Number of hits** | **Date** |
| --- | --- |
| 7 | 09.01.2020 |

### P

| [mh "Pancreatic Neoplasms"] OR  ("Pancreatic Neoplasm*"):ti,ab,kw OR  ("Pancreatic Cancer*"):ti,ab,kw OR  ("Pancreatic Tumor*"):ti,ab,kw OR  ("Pancreatic Tumour*"):ti,ab,kw OR  ("Pancreas Neoplasm*"):ti,ab,kw OR  ("Pancreas Cancer*"):ti,ab,kw OR  ("Pancreas Tumor*"):ti,ab,kw OR  ("Pancreas Tumour*"):ti,ab,kw OR  ("Cancer of Pancreas"):ti,ab,kw OR  ("Cancer of the Pancreas"):ti,ab,kw |  |
| --- | --- |

### I

| (Resection* OR  Resect* OR  Extirpation OR  Excision* OR  ectomy OR  removal):ti,ab,kw  AND  ([mh "Hepatic Artery"] OR  "Hepatic Arter*":ti,ab,kw OR  [mh "Mesenteric Artery, Superior"] OR  "Superior Mesenteric Arter*":ti,ab,kw OR  [mh "Celiac Artery"] OR  "Celiac Arter*":ti,ab,kw) |  |
| --- | --- |

## CINAHL

| **Number of hits** | **Date** |
| --- | --- |
| 63 | 09.01.2020 |

### P

| "Pancreatic Neoplasm*" OR  "Pancreatic Cancer*" OR  "Pancreatic Tumor*" OR  "Pancreatic Tumour*" OR  "Pancreas Neoplasm*" OR  "Pancreas Cancer*" OR  "Pancreas Tumor*" OR  "Pancreas Tumour*" OR  "Cancer of Pancreas" OR  "Cancer of the Pancreas" |  |
| --- | --- |

### I

| (Resect* OR  Extirpation OR  Excision* OR  ectomy OR  removal)  AND  ("Hepatic Arter*" OR  "Superior Mesenteric Arter*" OR  "Celiac Arter*") |  |
| --- | --- |

## Clinical Trial Gov

<http://www.clinicaltrials.gov/>

| **Number of hits** | **Date** |
| --- | --- |
| 23 | 09.01.2020 |

### P

| "Pancreas Karzinom" OR  "Pancreatic Neoplasm" OR  "Pancreatic Cancer" |  |
| --- | --- |

### I

| Resection AND  ("Hepatic Artery" OR  "Superior Mesenteric Artery" OR  "Celiac Artery") |  |
| --- | --- |

### Suchsätze

|  |  | **Trefferzahl** |
| --- | --- | --- |
| **P** | ("Pancreas Karzinom" OR "Pancreatic Neoplasm" OR "Pancreatic Cancer")  AND |  |
| **I** | (Resection AND ("Hepatic Artery" OR "Superior Mesenteric Artery" OR "Celiac Artery" )) |  |

## International Clinical Trials Registry Platform ICTRP (WHO Trials)

<http://www.who.int/ictrp/en/>

| **Number of hits** | **Date** |
| --- | --- |
| 3 | 09.01.2020 |

### P

| Pancreatic Neoplasm OR  Pancreatic Cancer OR  Pancreatic Tumor OR  Pancreas Neoplasm OR  Pancreas Cancer OR  Pancreas Tumor |  |
| --- | --- |

### I

| Resection AND Hepatic Artery OR  Resection AND Superior Mesenteric Artery OR  Resection AND Celiac Artery |  |
| --- | --- |

### Suchsätze

|  | **Suchsatz** | **Trefferzahl** |
| --- | --- | --- |
| **Condition** | Pancreatic Neoplasm OR Pancreatic Cancer OR Pancreatic Tumor OR Pancreas Neoplasm OR Pancreas Cancer OR Pancreas Tumor |  |
| **Intervention** | Resection AND Hepatic Artery OR Resection AND Superior Mesenteric Artery OR Resection AND Celiac Artery |  |
